# Weekday and seasonal patterns in psychiatric referrals in three major London A&E departments, 2012–2014

**DOI:** 10.1192/bjb.2017.4

**Published:** 2018-02

**Authors:** James Dove, Amit Mistry, Nomi Werbeloff, David Osborn, Nora Turjanski

**Affiliations:** 1 Camden & Islington NHS Foundation Trust, London; 2 Barnet, Enfield & Haringey Mental Health Trust, London; 3 Division of Psychiatry, UCL; 4 Division of Psychiatry, Faculty of Brain Sciences, UCL

## Abstract

**Aims and method:**

To identify temporal and demographic trends in referrals made to psychiatric liaison services. Routine clinical data from 16 105 individual referrals from three central London accident and emergency (A&E) departments to psychiatric liaison services from 2012 to 2014 were obtained and analysed using the Clinical Record Interactive Search (CRIS).

**Results:**

Referrals from A&E to psychiatric liaison services increased 16% over the 3-year study period. There were fewer referrals to psychiatric liaison services in winter months compared with other seasons. There were fewer referrals to psychiatric liaison services over the weekend compared with weekdays (average 15.4 daily weekday referrals *v*. 13.2 weekend, *z* = 5.1, *P* < 0.001), and weekend referrals were slightly less likely to result in admission to psychiatric hospital (11.3% *v*. 12.8%, respectively, χ^2^ = 6.33, *P* = 0.01).

**Clinical implications:**

Psychiatric staffing in A&E and inpatient psychiatric wards requires planning to meet temporal and regional variations in the pattern of demand.

**Declaration of interest:**

None.

Accident and emergency (A&E) departments in the UK are getting busier, with an estimated 50% increase in demand over the past 10 years.^1^ Approximately 5% of A&E attendances are for treatment of a mental disorder.^2^ An estimated 8% of all A&E attendances are by ‘chronic repeat attenders’; the most common reason for frequent attendance is an untreated mental health problem.^1^

The increased demands on A&E and the consequent impact on bed availability in acute trusts have been well studied, and a trend in peak pressures on A&E departments over the winter months has been established.^3^^,^^4^ Although it is acknowledged that A&E attendances are generally lower in the winter months, patients are likely to be more unwell, requiring admission that can lead to longer hospital stays and long waiting times in A&E – the ‘winter pressures’.^5^ This phenomenon has attracted injections of short-term funding of staff and resources in an attempt to encourage flow through the system – including to liaison mental health teams.

Public awareness of these pressures has led to a number of developments in recent years. These include the drive towards consultant-delivered care and, more recently, the ‘7-day National Health Service (NHS)’ proposals made by Bruce Keogh initially in 2013.^6^^,^^7^ These initiatives are now supported by government policy following suggestions that, further to the pressures on NHS services, there may be a weekend effect in terms of increased morbidity and mortality.^8^

A recent study by Patel *et al*^9^ investigated the mortality relating to *psychiatric* weekend admissions and found no correlation between weekend admission and increased mortality. However, the study identified that those admitted at the weekend had shorter admissions and higher readmission rates, leading to the suggestion that there is a different population who are more likely to be admitted at weekends. There is little or no work looking at the fluctuation in presentation at the gateways to psychiatric admissions – of which A&E attendance is a major source.

Anecdotally, it is said that ‘major’ mental illness, i.e. bipolar affective disorder and schizophreniform disorders, have bimodal peaks in presentation of spring and autumn;^10^^,^^11^ it might be assumed, therefore, that these would be peak admission times for psychiatric patients.

Here, we describe a study that analyses 3 years of referral data from three central London A&E departments to their psychiatric liaison teams, looking at seasonal variation and variation in weekday/weekend referrals, as well as some limited analysis of the demographics of those patients referred.

## Aims

The aim of this study is to describe referral rates from three central London A&E departments to their respective psychiatric liaison services, to explore whether patterns of referral are similar to trends reported regarding general attendance to A&E, including the weekend and ‘winter pressures’ models reported nationally. The secondary aim was to compare weekend *v*. weekday referral trends, and whether these referrals were more or less likely to result in admission to inpatient services. The tertiary aim was to assess those presenting frequently to A&E services (≥3 times during the study period), and determine whether these patterns were more pronounced in this group of patients.

## Method

Routine clinical data for this study was obtained from Camden & Islington (C&I) NHS Foundation Trust using the Clinical Record Interactive Search (CRIS) tool. CRIS is an application developed to enable routinely collected electronic health records to be used in research, using an explicit deidentification process.^12^ C&I is a large mental health provider serving a geographic catchment area of two inner-city London boroughs, and approximately 440 000 residents. The database contains full but anonymised information from over 100 000 mental health patients.^13^ Studies using CRIS received ethical approval from the National Research Ethics Service (NRES) Committee East of England – Cambridge Central (14/EE/0177).

We conducted a retrospective cohort study of all A&E referrals to psychiatric liaison services across three London teaching hospitals (Royal Free Hospital (RFH) and Whittington Hospital (WH) in North London, and University College London Hospital (UCLH) in Central London) over a 3-year period (2012–2014), deriving a complete data set of 16 105 individual psychiatric referrals. These 3 years were chosen since complete electronic record data was available for all three sites.

Data collection on the 16 105 referrals was limited to fields that are well recorded on CRIS and encompassed the following:

• day and date of referral from A&E to psychiatric liaison service
• demographic details of referee – age, gender, ethnicity
• discharge destination of referral, i.e. admission or discharge
• admission, i.e. informal or under a section of the Mental Health Act.

Data on diagnosis were not used for purposes of this study, as the majority of the patients referred to liaison teams are not allocated a recorded ICD-10 diagnosis, for instance, where no mental disorder is present after assessment.

Discharge destination of liaison referral being admission to psychiatric hospital was used as a ‘proxy of severity’ of presentation, in common with other similar studies.^14^^–^^20^

Number of presentations of individual patients during the time period was also recorded, and those presenting ≥3 times in the study period were identified as ‘frequent attenders’ and analysed as a separate cohort within the study in an attempt to identify any differences in patterns of referral for this group.

## Statistical analysis

Number of referrals per year was expressed as a proportion of referrals from the total population in the C&I catchment area (426 463 according to the 2011 census). Z-tests were used to compare the proportion of referrals between the different study years, seasons and days of the week (weekday *v*. weekend).

Descriptive statistics of all patients referred over the study period were examined.

The chi-square test of independence (χ^2^) was used to compare the number of referrals across seasons and days of the week.

Multilevel logistic regressions were used to account for multiple referrals of one patient and to examine the association between patient characteristics (sex, age and ethnicity) and weekend referral.

Finally, frequent attenders were compared to non-frequent attenders using the χ^2^ test for categorical variables and independent samples *t*-test for continuous variables.

## Results

In the years 2012–2014, there were a total of 16 105 referrals from A&E services to psychiatric liaison teams in the study area (RF: 4575, UCLH: 6440, WH: 5090). These referrals represent a total of 10 049 individual patients referred. The total number of referrals per patient varied from 1 to 49.

### Description of cohort

The average age of patients referred was 38.8 years (SD = 15.6); 92.6% were under 65 years of age. 51.2% of the cohort were male. Ethnicity data were missing for 17% of the sample. Of those with complete data, the majority of patients were of White ethnic origin (69.5%).

Frequent attenders (≥3 referrals over study period, *n* = 1108) did not differ from the rest of the cohort with regard to gender and age distribution, although there was a slightly higher proportion of people of White ethnic origin.

### Trend in referrals year on year

As can be seen in Table 1, there was an increase of 16% (*z* = 7.764 *P* < 0.001) in total referrals over the 3 years across the three sites (RF +36%, WH +7%, UCLH +12%).

**Table 1:** Total referrals to psychiatric liaison service from A&E by hospital site by year

Site	2012	2013	2014	Total % increase 2012–2014
Royal Free Hospital	1229	1679	1667	36%
Whittington Hospital	1606	1769	1715	7%
University College London Hospital	2076	2040	2324	12%
Total	4911	5488	5706	16%
Referrals as proportion of population in catchment area	1.15%	1.29%	1.34%	–

Over the 3 years, 12.4% of referrals led to an inpatient psychiatric admission (*n* = 2003), 33.4% of those (*n* = 654) under a section of the Mental Health Act.

### Seasonality of referrals

**Fig. 1:**
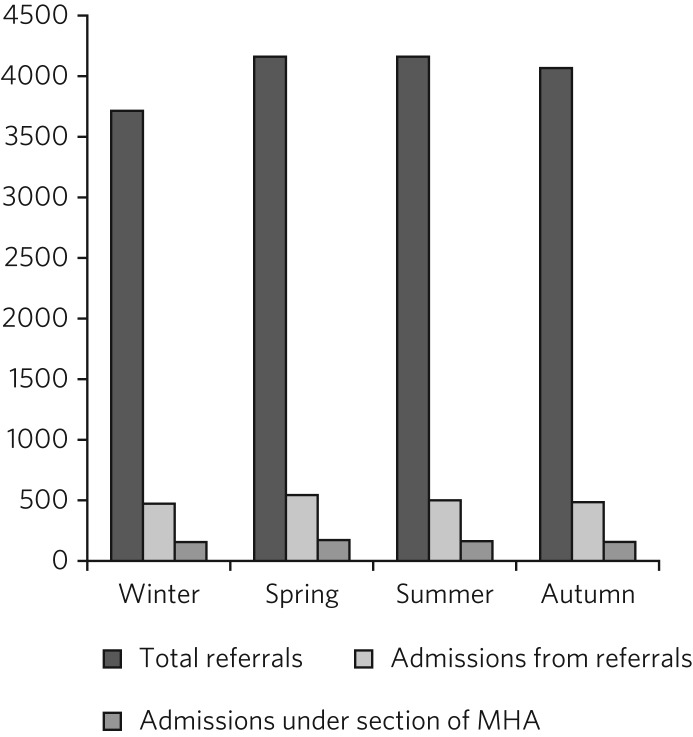
All referrals to psychiatric liaison services from A&E 2012–2014; comparison with subsequent admissions from those referrals, ‘informal’ and under a section of the Mental Health Act; grouped by season.

When the 16 105 referrals across the year were divided by season (defined as: winter, December to February; spring, March to May; Summer, June to August; Autumn, September to November) the only statistically significant finding was that, compared with all other seasons, the winter months saw fewer referrals (*z* = 4.8, *P* < 0.001; see Table 2 and Fig. 1). This matched with the lowest percentage overall of admissions from all seasons. Peak admissions were seen in the spring – 13.1% or 546 admissions over the 3 years – however, the percentage of referrals resulting in admission did not differ significantly by season (χ^2^ = 3.92, *P* = 0.27). Similarly, there was no statistically significant difference between the percentage of referrals resulting in admission under the Mental Health Act by season (χ^2^ = 0.30, *P* = 0.96).

**Table 2:** Comparison of referrals to psychiatric liaison service from A&E and subsequent admissions, by season

	Winter	Spring	Summer	Autumn	Total
Total no. of referrals (%)	3715 (23.1)	4160 (25.8)	4162 (25.8)	4068 (25.3)	16 105
Proportion of population (%)	0.87%	0.98%	0.98%	0.95%	
Admissions from referrals (%)	474 (12.8)	546 (13.1)	496 (11.9)	487 (12.0)	2003
No. of admissions under section of the Mental Health Act (%)	157 (33.1)	174 (31.8)	165 (33.3)	158 (32.4)	654

### Weekend referrals

Of the 16 105 referrals, there were fewer referrals to psychiatric liaison services at weekends compared with weekdays (0.48% *v*. 0.56% of the population; *z* = 5.1 *P* < 0.001).

Fewer weekend referrals resulted in inpatient admissions compared with weekday referrals (11.3% *v*. 12.8%, respectively, χ^2^ = 6.33, *P* = 0.01). Of the weekday referrals that resulted in admissions, 33.3% were under a section of the Mental Health Act. Of the weekend referrals that resulted in admissions, 30.6% were under a section. This difference is not statistically significant (χ^2^ = 1.20, *P* = 0.27).

Multilevel logistic regressions suggested that patients referred on weekends were more likely to be female, under the age of 65 and of White ethnic origin (Table 3).

**Table 3:** Comparison of demographic data of all referrals to psychiatric liaison services; weekend *v*. weekday attenders

	Total no. of referrals	Weekend referrals, %	Odds ratio (95% CI), *P*
Total referrals	16 105		
Gender			
Male	8330	24.8	Reference
Female	7775	26.6	1.11 (1.03–1.19), 0.006
Age			
Average (years)	39.36		
<65	15 049	26.2	Reference
>65	1056	23.7	0.87 (0.71–0.96), 0.015
Ethnicity^a^			
White	10 116	26.7	Reference
Asian	686	21.6	0.75 (0.62–0.91), 0.004
Black	1778	24.2	0.86 (0.76–0.98), 0.02
Other	1535	25.7	0.95 (0.83–1.07), 0.40

a. Data on ethnicity missing for 1726 (17.2%) participants.

## Discussion

Referrals to psychiatric liaison services between 2012 and 2014 within three central London A&E departments echoed the national figures for A&E attendances, with increased overall attendance year on year. Our results also showed a seasonal trend similar to the A&E data, with decreased absolute referrals in winter months. However, in contrast to the general hospital population, these referrals appear to be for people with a lower severity of illness in the winter months (using the proxy outcome measure of an admission to inpatient psychiatric services resulting from those referrals). Our data showed increased severity of presentations (increased admissions) occurring outside the winter months, but there was no statistically significant variation in number of patients admitted informally or under the Mental Health Act throughout the year.

Weekdays were slightly busier in terms of average numbers of psychiatric referrals and admissions than weekends, in terms of both numbers of referrals and numbers of admissions to psychiatric inpatient beds (11.3% *v*. 12.8%, respectively, χ^2^ = 6.33, *P* = 0.01).

There is only limited evidence from this data set to support the concept of a defined seasonal variation in psychiatric presentation; despite the academic position that ‘major’ mental illness – bipolar affective disorder and schizophreniform disorders – have bimodal peaks in presentation of spring and autumn.^1^ This phenomenon might, however, be able to explain the trend seen in our data of a shift in severity of illness when comparing the psychiatric population with the general acute hospital intake, with a peak of admission rates from winter to spring; however, there were no reliable data in this study on diagnosis.

This study demonstrates an increased presentation of mental health problems to A&E, and increased severity of those presentations, during the week rather than at the weekend.

Key points and implications for A&E and psychiatric liaison services from this study are as follows.

• There was a significant increase in number of referrals from A&E to psychiatric liaison services year on year.
• Winter was significantly different from the three other seasons (with lower referrals).
• There were significantly fewer referrals per day (on average) on weekend *v*. weekdays, but the absolute difference was only 1–2%.

### Limitations

We looked at referrals to mental health liaison services, rather than totals for A&E mental health presentations. It is anticipated that a far higher proportion of patients with a primary psychiatric reason for presentation are managed by A&E staff and discharged without referral to mental health liaison services. It could be argued, therefore, that referral itself could be used as a proxy for severity of presentation.

This study looks at only one route of psychiatric presentation – through A&E – and does not include other routes of presentation, i.e. crisis teams, general practitioner, etc., and it is therefore not a comprehensive picture of fluctuation in need throughout the year.

Diagnosis was not reliably recorded in the data set and therefore not included in this study – a major limitation in discussion around seasonal variations in psychiatric illness presentation.

We have no data on timings of referrals and we are therefore unable to comment on ‘out of hours’ attendance other than weekday/weekend comparisons.

It is highly likely that there is a variety in threshold for referral between sites and at different times of year. For example, higher absolute summer referrals could possibly be accounted for by an influx of new doctors with lower thresholds for referral, resulting in an increase in summer referral rates but lower severity of presentation; however, we have used statistical tests in the data set in an attempt to mitigate the impact of these variables.

Use of psychiatric admission as a proxy for severity is not without its limitations: decisions to admit, particularly informally, may well be linked to bed pressures, abilities of home treatment teams locally, etc. The admissions under a section of the Mental Health Act should be less susceptible to these variables.

Although there are differences in the populations that the three hospitals serve, they are of similar size and location with equally diverse local populations, allowing for a good generalisability of the data. The e-record (RiO) is the only records system used by the psychiatric teams at all three sites and as such is a reliable representation of all patients seen.

## About the authors

**James Dove** is an ST5 Psychiatric Trainee at Camden & Islington NHS Foundation Trust, London, UK. **Amit Mistry** is an ST5 Psychiatric Trainee at Barnet Enfield & Harringey Mental Health Trust, London, UK. **Nomi Werbeloff** is a PhD Research Associate at UCL, and Camden & Islington NHS Foundation Trust. **David Osborn** is a Professor of Psychiatric Epidemiology at Division of Psychiatry, Faculty of Brain Sciences, UCL and a Consultant Psychiatrist at Camden & Islington NHS Foundation Trust. **Nora Turjanski** is a Consultant Liaison Psychiatrist at Camden & Islington NHS Foundation Trust.
